# Fibrodysplasia ossificans progressiva in a young adult with genetic mutation: Case report

**DOI:** 10.1097/MD.0000000000024620

**Published:** 2021-03-05

**Authors:** Zhankui Wang, Xiuhua Wang, Baojin Liu, Yanfeng Hou

**Affiliations:** a Department of Rheumatology, The First Affiliated Hospital of Shandong First Medical University, Jinan, Shandong; b Shandong Provincial Key Laboratory for Rheumatic Disease and Translational Medicine, Jinan; c Shandong First Medical University, Jinan, Shandong, China.

**Keywords:** diagnosis, fibrodysplasia ossificans progressiva, gene

## Abstract

**Rationale:**

Fibrodysplasia ossificans progressiva (FOP) is a rare autosomal dominant disorder characterized by congenital skeletal deformities and soft tissue masses that progress into heterotopic ossification. Deformities of the great toes are distinctive and heterotrophic ossification usually begins in the first decade of the patient's life. Any invasive procedure could potentially trigger a flare and heterotopic calcification. The diagnosis is difficult and there is no effective treatment for FOP and the approximate life expectancy is 4 decades.

**Patient concerns:**

A 22-year-old male patient who had suffered from pain and movement limitations for 14 years. At the early stage of disease, the child underwent an operation on both thighs with a diagnosis of myophagism. He had serious stiffness and multiple bony masses with the characteristic bilateral hallux valgus deformity and microdactyly.

**Diagnoses:**

The patient was diagnosed with FOP by the help of characteristic great toe malformations and widespread heterotopic ossification throughout the body. Deoxyribonucleic acid sequencing demonstrated that the patient had a de novo heterozygous mutation (c.617G>A; p.R206H) in activin A receptor/activin-like kinase 2.

**Interventions:**

We administered a co-therapy of glucocorticoids, NSAIDs to relieve pain, and montelukast for 2 months. Bisphosphonate (5 mg, intravenous) was used once.

**Outcomes:**

At the follow-up 12 months later, the patient still felt low back pain sometimes and need take NSAIDs three times a week.

**Lessons:**

Clinicians and radiologists should realize the characteristic features of FOP and early diagnosis can prevent additional invasive harm to the patient.

## 1 Introduction

Fibrodysplasia ossificans progressiva (FOP) is a very rare autosomal dominant disorder characterized by ectopic osteogenesis in connective tissue. Its worldwide prevalence is estimated to be 1 case per 2 million individuals.^[1]^ Recently, it was shown that heterozygous activating mutations in the activin A receptor, type I/activin-like kinase 2 (ACVR1/ALK2) are primarily responsible for this disease.^[2]^ Ossification commonly begins in the first decade of life, ranging from 6 months to 13 years.^[3]^ The main clinical features of FOP are malformations of the great toes and painful soft-tissue swelling that eventually progresses to heterotopic ossification. Misdiagnosis rates could be up to 90%,^[4]^ and these patients may undergo invasive diagnostic procedures that can trigger a flare-up and lead to permanent harm. There is no effective treatment for FOP, and the prognosis is poor. The approximate life expectancy is 4 decades. Common causes of death are secondary to cardiopulmonary complications.^[5]^

In the current case report, we describe a 22-year-old man patient with the classic clinical manifestations of FOP, but he was misdiagnosed for many years. We performed deoxyribonucleic acid sequencing analysis on this patient and confirm the diagnosis of FOP.

## 2 Case report

A 22-year-old male was admitted to our outpatient department with complaints of multiple swellings and stiffness in the back and both arms and legs for the past 14 years. At the age of 8, the patient developed slight stiffness in both thighs. It was difficult for him to squat, and his restriction in movements persisted. At the age of 10, the child underwent an operation on both thighs at the local hospital with a diagnosis of myophagism. There was no apparent improvement, and the stiffness began to spread, with diffuse swelling and discrete lumps on the upper and lower back and a limited range of motion of the neck and shoulders. Three years ago, a gradually increasing, painless swelling on the lateral aspect of his right distal arm was associated with restricted extension of the right elbow joint. He had difficulty feeding himself by his right hand and with dressing and undressing himself. Three months ago, a gradually increasing painful swelling was noticed on his sacrococcygeal region.

He was the first child of healthy, nonconsanguineous parents and had a healthy 20-year-old sister. He remembered that his grandmother had microdactyly of the hallux.

**Figure 1:**
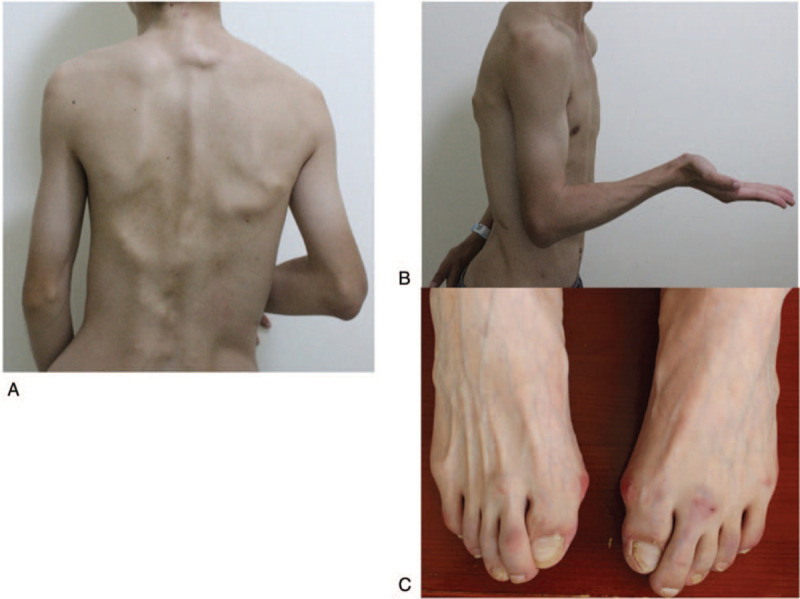
(A) Multiple irregular, nontender, bony hard swellings were found on the neck, scapular, and lumbar regions, extending up to the lower sacrococcygeal region. (B) Multiple bony masses of irregular sizes on the right lateral forearm and the metacarpal aspect of the ipsilateral wrist were associated with a fixed flexion deformity of 90° at the right elbow joints and a restriction of wrist movement. (C) Halluces were characterized by hallux valgus deformity with macrodactyly.

On physical examination, multiple irregular, nontender, bony hard swellings were found on the neck, scapular, and lumbar regions, extending up to the lower sacrococcygeal region (Fig. 1A). He had limited rotation of the neck with limited abduction of the shoulders. Multiple bony masses of irregular sizes on the right lateral forearm and the metacarpal aspect of the ipsilateral wrist were associated with a fixed flexion deformity of 90° at the right elbow joints and a restriction of wrist movement (Fig. 1B). In addition, both halluces were characterized by hallux valgus deformity with microdactyly (Fig. 1C).

**Figure 2:**
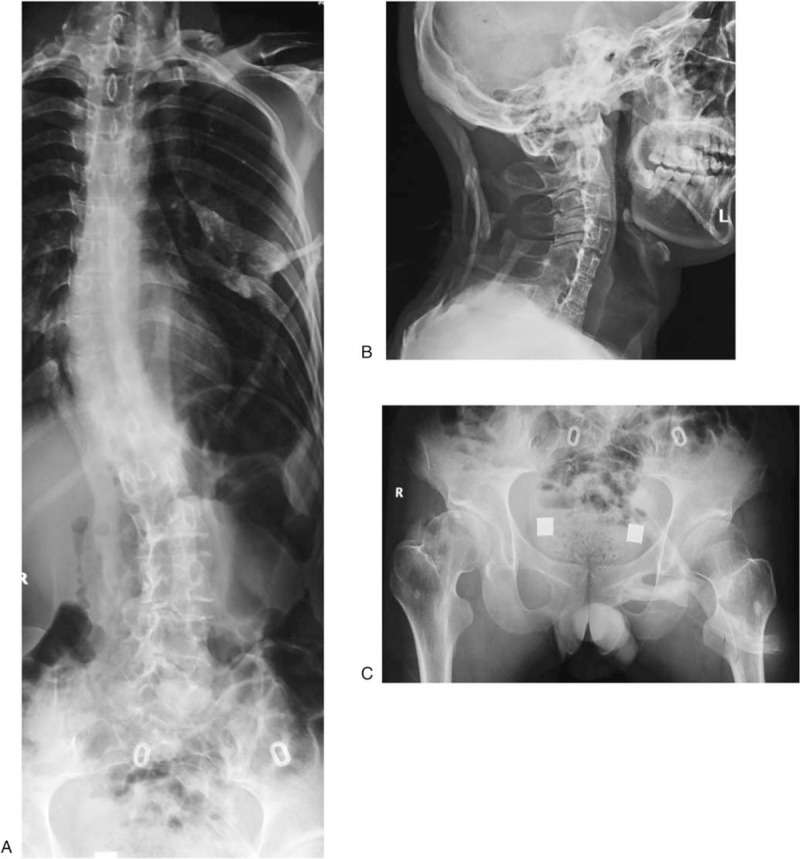
(A) X-ray and magnetic resonance imaging showed fusion anomalies of heterotrophic ossification at the posterior elements of the cervical and upper thoracic spine, scapula, lumbar paraspinal muscle area, and hip area. (B) Lateral cervical spine radiography demonstrated osseous bridging of the posterior elements. (C) Anteroposterior hip X-ray showed a short, broad femoral neck on both sides with bridge-like heterotopic ossifications extending across the left hip joints and iliac bone as well as lateral to the thigh.

All laboratory tests, including acute phase reactants, bone markers, muscle enzymes, and all other biochemical parameters, were normal. X-ray and magnetic resonance imaging showed fusion anomalies of heterotrophic ossification at the posterior elements of the cervical and upper thoracic spine, scapula, lumbar paraspinal muscle area, and hip area (Fig. 2A). Lateral cervical spine radiography demonstrated osseous bridging of the posterior elements (Fig. 2B). Anteroposterior hip X-ray showed a short, broad femoral neck on both sides with bridge-like heterotopic ossifications extending across the left hip joints and iliac bone as well as lateral to the thigh (Fig. 2C). The results of genetic testing showed that the patient had a heterozygous mutation (c.617G>A; p.R206H) in ACVR1/ALK2. Thus, the patient was diagnosed with FOP when the canonical ACVR1/ALK2 c.617 G>A (p.R206H) mutation was detected.

He was treated with glucocorticoids (methylprednisolone, 40 mg/d for 4 days at the beginning) with slow tapering of the corticosteroids, NSAIDs (meloxicam 7.5 mg/d) to relieve pain, and montelukast (10 mg/d, peroral) for 2 months. Bisphosphonate (5 mg, intravenous) was used once. Furthermore, we advised the patient to avoid trauma and surgery. After these treatments, the condition did not improve significantly. At the follow-up 12 months later, the patient still felt low back pain sometimes and need take NSAIDs 3 times a week. In addition, new swellings continued to appear on the right back with progressive ossification and restriction of right elbow movement. We advised the patient to take immunosuppressive agents such as methotrexate or cyclosporine. Be afraid of the side effects of the drugs, he is still considering.

## 3 Discussion

FOP is a rare autosomal dominant disorder characterized by congenital skeletal deformities and soft tissue masses that progress into heterotopic ossification. Heterotrophic ossification usually begins in the first decade of the patient's life. There is no ethnic, racial, gender, or geographic predisposition.^[6,7]^ In fact, flare-ups of FOP and new ossifications can be initiated or triggered by trauma, viral infections, dental procedures, surgery, or vaccinations.^[8,9]^ Therefore, these events should be avoided after diagnosis.^[10]^ Therefore, it is important for clinicians to know about the features of FOP. The classic features of FOP have 2 specific physical phenotypes^[11]^: the presence of congenital hallux valgus with microdactyly and the formation of progressive heterotopic ossifications following a particular anatomic pattern. Most patients first present in early childhood with the development of soft-tissue swelling involving the back, axial skeleton, head, and proximal areas of the body that later reaches the ventral limbs, tailbone, and distal areas. The patient with FOP may experience a flare-up of the progression of the disease if they undergo invasive examination and treatment. Our patient also suffered from an invasive operation at the early stages of the disease, which triggered a rapid increase in other soft-tissue ossified masses. Before ossification, congenital deformities such as malformed great toes or hallux valgus are the earliest clinical manifestation that refer the patient to make a genetic analysis. However, great toe malformations are pathognomonic and do not occur in all patients.^[12]^ Other anomalies, such as a broad, short femoral neck, clinodactyly, thumb malformations, kidney stones, inverted nipples, cervical spine fusions, and conductive hearing impairment, can also be observed in FOP patients.^[13]^ The diaphragm, tongue, and extraocular, cardiac, and smooth muscles have not been found to be affected in patients with FOP that have been reported.

In this patient, bilateral short, broad femoral neck, and cervical spinal fusion were also detected by X-ray. Plain radiographs can identify the deformities of the high-density ossification of soft tissue and can also identify bony bridges in different fascial planes in the later stage of lesions. Computed tomography scans show high-density calcification foci in the muscles and partial fusion with adjacent bone. Magnetic resonance imaging can reveal lesions that may present as edema of the fascial planes and muscular bundles in the early stages of disease.^[3,14]^

The FOP-related ACVR1 gene coding bone morphogenetic protein type-1 receptor is located on the 2q23-24 chromosome. Skeletal muscle and cartilage express ACVR1 in many other tissues of the body. It has been confirmed that the most common mutation site is the same heterozygous missense mutation in the glycine–serine activation domain (c.617G>A; R206H) of ACVR1. Other sites include L196P, R258S, P197/F198L, R202I, Q207E, G325A, G328W/ G328E/G328R, G356D, R375P.^[15–18]^ Some patients with atypical mutations can have more severe presentations.^[2]^ The genetic breakthrough of inducing abnormal activation of bone morphogenetic proteins as the cause of FOP lesions established a critical milestone in our understanding FOP, and open the doors to find a highly conserved therapeutic target for the disease.^[11]^ The confirmatory genetic mutation testing of ACVR1/ALK2 is crucial to confirm a diagnosis of FOP before the appearance of heterotopic ossifications.

Haga et al drafted diagnostic criteria of FOP,^[19]^ the definite patient should meet not only clinical and radiological findings, but also definitive genetic confirmation (ACVR1 gene mutation). This patient meets all the conditions in the diagnostic criteria.

After diagnosis, there is no effective treatment for FOP to our current knowledge. Guidelines have been published that indicate that the use of glucocorticoids can manage new flare-ups affecting the function of major joints in the appendicular skeleton. During or after flare-up periods, NSAIDs, mast cell inhibitors, or narcotic analgesics can also be used. However, there is currently no proof that any therapy is effective in altering the natural history of the disease. Li et al treated 17 children with NSAIDs, glucocorticoids, and immunosuppressive agents. The immunosuppressive agents used included methotrexate and cyclosporine. The disease condition was controlled and improved.^[20]^ With an understanding of the mechanism of this specific FOP-causing gene mutation and an emerging understanding of the pathology of FOP, many studies have been performed on small-molecule biological agents for FOP.^[21]^ As reported in the literature, the median age of survival is approximately four decades. Most patients with FOP are usually dependent on a wheelchair by the third decade of life. Common causes of death for this disease are thoracic insufficiency syndrome or other pulmonary complications.^[22]^

In summary, the aim of this case-based review was to increase the awareness of this easily diagnosed disease so as to avoid invasive examination, which can lead to the deterioration of the patient's condition.

## Author contributions

**Conceptualization:** Yan Feng Hou.

**Data curation:** Yan Feng Hou.

**Funding acquisition:** Xiuhua Wang, Baojin Liu.

**Investigation:** Xiuhua Wang.

**Methodology:** Xiuhua Wang.

**Visualization:** Zhankui Wang.

**Writing – original draft:** Zhankui Wang.

**Writing – review & editing:** Baojin Liu.

## References

[1] Kaplan FS Xu M Glaser DL. Early diagnosis of fibrodysplasia ossificans progressiva. Pediatrics 2008;121:e1295–300. 18450872 10.1542/peds.2007-1980 PMC3502043

[2] Kaplan FS Xu M Seemann P. Classic and atypical fibrodysplasia ossificans progressiva (FOP) phenotypes are caused by mutations in the bone morphogenetic protein (BMP) type I receptor ACVR1. Hum Mutat 2009;30:379–90. 19085907 10.1002/humu.20868 PMC2921861

[3] Bauer AH Bonham J Gutierrez L. Fibrodysplasia ossificans progressiva: a current review of imaging findings. Skeletal Radiol 2018;47:1043–50. 29445932 10.1007/s00256-018-2889-5

[4] Kitterman JA Kantanie S Rocke DM. Iatrogenic harm caused by diagnostic errors in fibrodysplasia ossificans progressiva. Pediatrics 2005;116:e654–661. 16230464 10.1542/peds.2005-0469

[5] Sun Y Xia W Jiang Y. A recurrent mutation c.617G>A in the ACVR1 gene causes fibrodysplasia ossificans progressiva in two Chinese patients. Calcif Tissue Int 2009;84:361–5. 19300893 10.1007/s00223-009-9235-9

[6] Morales-Piga A Kaplan FS. Osteochondral diseases and fibrodysplasia ossificans progressiva. Adv Exp Med Biol 2010;686:335–48. 20824454 10.1007/978-90-481-9485-8_19 PMC4913786

[7] Shore EM Feldman GJ Xu M. The genetics of fibrodysplasia ossificans progressiva. Clin Rev Bone Miner Metab 2005;3:201–4.

[8] Scarlett RF Rocke DM Kantanie S. Influenza-like viral illnesses and flare-ups of fibrodysplasia ossificans progressiva. Best Pract Res Clin Rheumatol 2004;275–9. 10.1097/01.blo.0000129557.38803.26 15232462

[9] Civan M Bilgili F Kilic A. A case of fibrodysplasia ossificans progressiva in a 5-year-old boy with all musculoskeletal features and review of the literature. J Orthop Case Rep 2018;8:36–9. 10.13107/jocr.2250-0685.1200 PMC6367301 30740372

[10] Shore EM Xu M Feldman GJ. A recurrent mutation in the BMP type I receptor ACVR1 causes inherited and sporadic fibrodysplasia ossificans progressiva. Nat Genet 2006;38:525–7. 16642017 10.1038/ng1783

[11] Robert J Pignolo Eileen M. Fibrodysplasia ossificans progressiva: diagnosis, management, and therapeutic horizons. Pediatr Endocrinol Rev 2013;10 Suppl 2:437–48. 23858627 PMC3995352

[12] Eresen Yazicioglu C Karatosun V Kizildag S. ACVR1 gene mutations in four Turkish patients diagnosed as fibrodysplasia ossificans progressiva. Gene 2013;515:444–6. 23260810 10.1016/j.gene.2012.12.005

[13] Morales-Piga A Bachiller-Corral J Gonzalez-Herranz P. Osteochondromas in fibrodysplasia ossificans progressiva: a widespread trait with a streaking but overlooked appearance when arising at femoral bone end. Rheumatol Int 2015;35:1759–67. 26049728 10.1007/s00296-015-3301-6

[14] Daniel Solomon Iyasu Wakjira Daniel Hailu. Fibroplasia ossificans progressiva: a case report of a rare disease entity. Ethiop J Health Sci 2018;28:513–6. 30607064 10.4314/ejhs.v28i4.17 PMC6308737

[15] Bocciardi R Bordo D Di Duca M. Mutational analysis of the ACVR1 gene in Italian patients affected with fibrodysplasia ossificans progressiva: confirmations and advancements. Eur J Hum Genet 2009;17:311–8. 18830232 10.1038/ejhg.2008.178 PMC2986177

[16] Gregson CL Hollingworth P Williams M. A novel ACVR1 mutation in the glycine/serine-rich domain found in the most benign case of a fibrodysplasia ossificans progressiva variant reported to date. Bone 2011;48:654–8. 21044902 10.1016/j.bone.2010.10.164 PMC3160462

[17] Whyte MP Wenkert D Demertzis JL. Fibrodysplasia ossificans progressiva: middle-age onset of heterotopic ossification from a unique missense mutation (c.974G>C, p.G325A) in ACVR1. J Bone Miner Res 2012;27:729–37. 22131272 10.1002/jbmr.1473

[18] Petrie KA Lee WH Bullock AN. Novel mutations in ACVR1 result in atypical features in two fibrodysplasia ossificans progressiva patients. PLoS One 2009;4:e5005. 19330033 10.1371/journal.pone.0005005 PMC2658887

[19] Haga N Nakashima Y Kitoh H. Fibrodysplasia ossificans progressiva: review and research activities in Japan. Pediatr Int 2020;62:3–13. 31774601 10.1111/ped.14065

[20] Zhang JM Li CF Ke SY. Analysis of clinical manifestations and treatment in 26 children with fibrodysplasia ossificans progressiva in China. World J Pediatr 2019;16:82–8. 31529313 10.1007/s12519-019-00302-x

[21] Cappato S Giacopelli F Ravazzolo R. The horizon of a therapy for rare genetic diseases: a “druggable” future for fibrodysplasia ossificans progressiva. Int J Mol Sci 2018;19:989. 10.3390/ijms19040989 PMC5979309 29587443

[22] Kaplan FS Zasloff MA Kitterman JA. Early mortality and cardiorespiratory failure in patients with fibrodysplasia ossificans progressiva. J Bone Joint Surg Am 2010;92:686–91. 20194327 10.2106/JBJS.I.00705 PMC2827822

